# Knowledge, Awareness, and Attitudes Towards HPV and Its Vaccination Among Women in the Medina Region: A Cross-Sectional Study

**DOI:** 10.3390/healthcare12232339

**Published:** 2024-11-23

**Authors:** Muhammad Abubaker Tobaiqi, Rawaf Adel Albouq, Ahmad Mustafa Ban, Abeer Khalid Alharbi, Razan Abdulrahman Alhejaili, Hanan Mohammed Alrefaei, Atheer Mubarak Alahmadi, Sara Mohammed Jaan, Asmaa Abdulmajeed Alshinqiti, Shahad Ali Alraddadi, Abdulaziz Ali Alraddadi, Fahad M. Altowairqi, Ibrahim S. Almalki, Muayad Albadrani

**Affiliations:** 1 Department of Family and Community Medicine and Medical Education, College of Medicine, Taibah University, Madinah 42353, Saudi Arabia; 2 College of Medicine, Taibah University, Madinah 42353, Saudi Arabia; 3 College of Medicine, Alrayan Colleges, Madinah 42541, Saudi Arabia; 4 Yanbu General Hospital, Madinah Health Cluster, Yanbu 46421, Saudi Arabia; 5 Royal Saudi Land Forces Medical Administration, Madinah Regional Command, Madinah 42375, Saudi Arabia; 6 Preventive Medicine Department, Prince Sultan Armed Forces Hospital in Madinah, Madinah 42375, Saudi Arabia

**Keywords:** HPV, human papillomavirus, Saudi Arabia, Medina

## Abstract

Background: HPV represents one of the most common sexually transmitted infections worldwide, with significant adverse clinical consequences such as cervical cancer. However, the knowledge and awareness regarding HPV and its vaccination, particularly among Saudi women, are still under debate. Our study aims to investigate the knowledge, awareness, and attitude toward HPV and its vaccines among women in the Medina region. Methods: A descriptive cross-sectional study was conducted from July 2024 to September 2024 in women aged more than 18 years and residing in Medina. The collected data included information regarding women’s demographics, knowledge, awareness, and attitudes toward HPV and its vaccination. All the statistical analyses were executed using SPSS. Results: A total of 721 participants enrolled in our study after adequate completion of the online questionnaire. The majority of the population (45.2%) is aged 18–25 years, with 51.3% of participants being single regarding marital status. A total of 59.4% of the participants have heard about HPV, 37% know that it is sexually transmitted, and 37.4% know about its ability to cause cervical cancer. Social media and the internet were the primary sources of information regarding the HPV vaccine, with 41.6% thinking that the HPV vaccine can be effective against HPV infection. Around 40% of the participants showed an attitude toward receiving the HPV vaccine as they feel they are at risk. The educational status and monthly income were notably associated with the overall knowledge and awareness regarding HPV and its vaccine. Conclusions: Our descriptive cross-sectional study highlights the significant gap in knowledge and awareness regarding HPV and its vaccine, with a lack of awareness identified as the commonest barrier preventing people from receiving the HPV vaccination. Hence, enhancing the knowledge and awareness level is essential to increase vaccination rates.

## 1. Introduction

Human papillomavirus (HPV) infection is the most common sexually transmitted disease (STD). Over 75% of sexually active individuals are infected with different subtypes of HPV throughout their lives. Presentations include laryngeal warts (HPV-11), anogenital warts (HPV-6), and cervical carcinomas (HPV 16, 18) [1].

Over 99% of cervical cancers are linked to cancerous subtypes of HPV [2], specifically HPV subtypes 16 and 18 [1,3]. Cervical cancer is the fourth most common cancer in women and fourth most common cause of cancer-related mortality [4,5]. The annual incidences and morbidity related to cervical carcinoma are estimated to be around 500,000 and 300,000, respectively [6]. In Saudi Arabia, cervical cancer is the third most common cancer affecting women, prevailing in around 5 in every 100,000 women [7].

In the Middle East and North Africa (MENA) region, the prevalence of HPV varies significantly. For instance, in North Africa, HPV prevalence is around 23%, while in the Gulf Cooperation Council (GCC) countries, including Saudi Arabia, it is approximately 17% [8]. The prevalence of HPV in the Levant region is about 20% [8]. These figures highlight the regional variations and the need for targeted public health interventions.

In comparison, the European Union (EU) reports around 33,000 cases of cervical cancer annually, with 15,000 deaths. The USA has an estimated 42.5 million people living with HPV, with around 47,984 new HPV-associated cancer cases reported each year. These statistics underscore the global burden of HPV and the importance of vaccination programs.

Vaccination against HPV is the most established method for the prevention of HPV infections [9]. It prevents around 90% of cervical cancer incidences [3]. Currently, bivalent forms of the vaccines specifically induce immunity against HPV subtypes 16 and 18, which are the ones that cause cervical cancer [10]. Quadrivalent vaccines induce immunity against HPV subtypes 6 and 11, which cause anogenital and laryngeal warts, respectively [11]. The primary target of the vaccination is females aged 9–14 years, and it is also suitable for administration to females who are 15 or older [12].

Saudi Arabia implemented HPV vaccination in the national immunization program in 2018 [13]. The available vaccines include Cervarix, a bivalent vaccine targeting HPV types 16 and 18; and Gardasil, a quadrivalent vaccine targeting HPV types 6, 11, 16, and 18. These vaccines are available in some private hospitals and clinics, as well as during routine office visits in family medicine and pediatric clinics [14].

However, the knowledge, awareness, and attitude of Saudis toward HPV and its vaccines are still suboptimal. Algaadi and colleagues conducted a prior cross-sectional study that enrolled 516 participants, primarily residing in the central region of Saudi Arabia [15]. They found that 56.3% of the participants had poor knowledge regarding HPV vaccines. Moreover, only 35.9% of the participants knew that HPV is sexually transmitted, and 41.5% of them knew that it can cause cervical cancer. Several factors influence this negative attitude toward the HPV vaccine, including cultural and religious beliefs. Additionally, parental consent significantly influences the vaccination of the target group of girls [13,16,17,18,19].

To our knowledge, no prior cross-sectional study was conducted in Medina. As the community of Medina might share some beliefs that hinder adherence to the HPV vaccine, we aimed to conduct this cross-sectional study targeting females residing in Medina. We want to evaluate and identify the level of our population’s knowledge regarding HPV and its vaccine and assess the barriers and factors that affect their attitude toward HPV vaccination.

## 2. Methods

### 2.1. Study Design

We conducted this descriptive cross-sectional study assessing the knowledge, attitudes, and awareness of females in Medina toward HPV infection and their acceptance of the HPV vaccine. We reported this cross-sectional study following the instructions provided by the Strengthening the Reporting of Observational Studies in Epidemiology (STROBE) [20].

### 2.2. Inclusion and Exclusion Criteria

Our study included females who resided in Medina if they matched the following criteria: age of at least 18, willingness to participate, and willingness to complete the questionnaire. Exclusion criteria were age less than 18, males, incomplete questionnaire, and people who neither resided in Medina nor were willing to complete the questionnaire.

### 2.3. Study Instrument

We conducted this study based on an online questionnaire adopted from a prior relevant study. It was shared online, targeting the eligible females residing in Medina. The calculated sample size with a 95% confidence interval was 385 participants. Data collection continued from August 2024 to October 2024, exceeding the sample size calculator specified target sample.

The collected data through the first part of the questionnaire focused on specifying these participants’ characteristics: age, marital status, nationality, level of education, occupational status, region in Medina, and monthly family income. Then, we categorized our questionnaire into measures of knowledge and awareness about HPV (five questions), measures of knowledge toward HPV vaccination (11 questions), awareness of HPV vaccination (three questions), and attitude toward HPV vaccination (13 questions). Each participant was considered to have a good knowledge or awareness of HPV or its vaccination if they answered more than 50% of the questions correctly.

### 2.4. Statistical Analysis

The Statistical Package for Social Sciences (SPSS) version 26 was used to analyze this study. The participant’s demographics were summarized using descriptive statistics. Inferential statistics, including the chi-square or Fisher’s exact test, were used to derive associations between knowledge, awareness, or attitudes regarding HPV or its vaccination and patients’ demographics.

### 2.5. Ethical Considerations

The research committee at the General Directorate of Health Affairs in Madinah provided ethical approval for our questionnaire (IRB: 24-076). Additionally, informed consent containing a detailed study objective was provided to each participant, with collected data being used only for the study purposes.

## 3. Results

### 3.1. Participant Characteristics

Our studies enrolled a total of 721 participants who completed our online questionnaire. Most participants were aged 18–25 (326; 45.2%), followed by those aged between 26 and 30 (77; 10.7%). We divided subsequent age groups by five years; each roughly enrolled around 9% of the participants. Married participants represented 41.5% of the study population. Over 95% of participants were Saudis, around 70% had a bachelor’s degree, and most were either students or unemployed. The detailed characteristics of the participants are exhibited in Table 1.

### 3.2. Knowledge and Awareness of Participants Towards HPV

**Figure 1:**
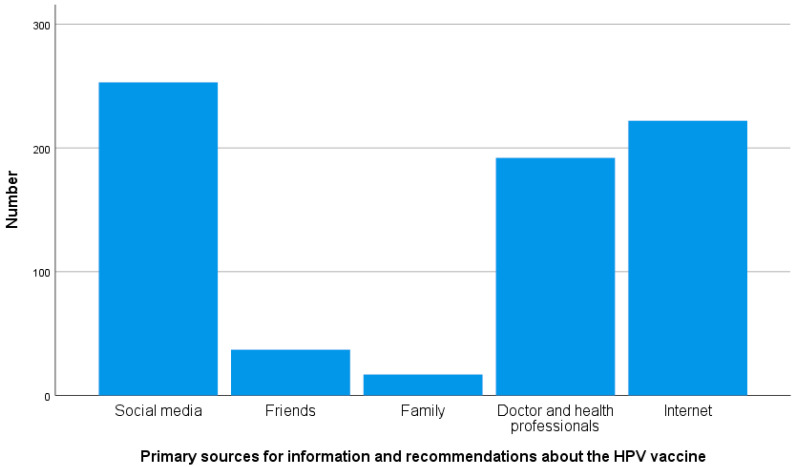
Primary sources for information and recommendations about the HPV vaccination.

The collected data showed that only 60% of the participants heard about HPV before this study. In addition, around 37% of participants knew that HPV is sexually transmitted and causes cervical cancer. Interestingly, less than 30% of the participants answered that doctors and health professionals should be the primary sources of information regarding HPV and its vaccination (Figure 1). Table 2 provides more details.

### 3.3. Knowledge and Awareness of Participants Towards HPV Vaccination

The collected data showed that 54.4% of the participants had heard about the HPV vaccine. Three hundred participants knew the effectiveness of vaccines against HPV. Around 32% believed that vaccines could protect against cervical cancer. Interestingly, less than 50% of the participants knew that HPV vaccines were available in Saudi Arabia. Moreover, around 50% of the participants had an incorrect insight that females should be screened for HPV before vaccination, and only 18% of the participants knew the schedule of HPV vaccination. Details regarding the knowledge and awareness of the participants toward HPV vaccines are presented in Table 2.

### 3.4. Attitudes Towards HPV Vaccination

Around 40% of the participants chose neutral as an answer to all questions assessing their attitude toward HPV vaccination. Additionally, 43% of participants strongly agreed with the need for more information regarding HPV vaccines before taking them. Consistently, roughly 60% of the participants indicated that lack of awareness regarding HPV and its vaccines is the most significant barrier to the administration of HPV vaccines. Concerns about vaccine safety also play an essential role, with 29.4% of respondents expressing this fear. Additionally, 17.5% of individuals are deterred by a fear of needles or injections. Time constraints affect 15.3% of participants, while 10.3% struggle with access to healthcare facilities. Cultural or religious beliefs (5.4%), family refusal (7.1%), and the cost of the vaccine (4.2%) are other notable barriers. A small percentage of respondents (1.8%) mentioned other reasons. The detailed attitude of participants regarding HPV vaccination is presented in Table 3.

### 3.5. Association Between Participants’ Demographics and Overall Knowledge and Awareness Level About HPV and HPV Vaccination

**Figure 2:**
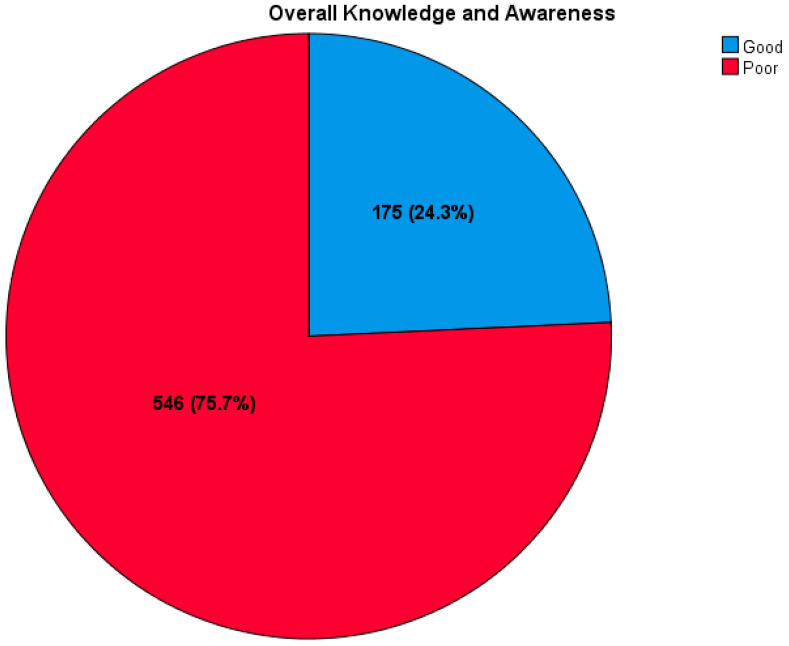
Frequency of overall knowledge and awareness of HPV and its vaccination.

Only 175 participants (24.3%) had good overall knowledge and awareness about HPV and HPV vaccination (Figure 2). Moreover, we found that participants’ level of education and monthly family income had a significant association with the concomitant knowledge and awareness level about HPV and its vaccination (*p* = 0.002 and <0.0001, respectively). Regarding participants’ level of education, comparable levels of good and poor knowledge and awareness were found among individuals with a bachelor’s degree (68% for both). However, more participants with a master’s or doctoral degrees had good knowledge and awareness (8.6% vs. 4.8% and 4.0% vs. 0.4%, respectively). Further data are exhibited in Table 4.

## 4. Discussion

In Medina, Saudi Arabia, the female population over 18 years old is approximately 400,000. This is based on the overall female population and age distribution data available for the city. Our study comprised 721 female participants, with 45.2% aged 18–25 years and 51.3% being single. The majority of women were Saudi (95.1%), 68% had a bachelor’s degree, and 33.6% had a monthly income ranging from 3000 to 10,000 RS/month. A total of 59.4% of the participants had heard about HPV, and only 37% knew that it was sexually transmitted. This demographic profile is somewhat representative of the young, educated female population in urban Saudi Arabia, but it may not fully represent the broader Saudi female population, particularly those in rural areas or with lower educational levels.

Our study highlights the significant gap in knowledge and awareness about HPV and its vaccination among women in the Medina region. The relatively low levels of awareness and knowledge observed in our study can be attributed to several factors, including cultural and religious beliefs, limited access to accurate information, and the influence of social norms. The high reliance on social media and the internet as primary sources of information underscores the need for reliable and accurate health information dissemination through these channels. The association between higher levels of education and better knowledge and awareness about HPV and its vaccination suggests that educational interventions could be effective at improving awareness and vaccination rates. Additionally, the significant association between monthly family income and knowledge and awareness indicates that socioeconomic factors play a crucial role in health education and access to vaccination. The barriers to HPV vaccination identified in our study, such as concerns about vaccine safety, fear of needles, and cultural or religious beliefs, highlight the need for targeted interventions to address these issues. Healthcare providers should be trained to effectively communicate the benefits and safety of HPV vaccination, and culturally sensitive educational campaigns should be developed to address misconceptions and promote vaccination. Our findings are consistent with previous studies conducted in other regions of Saudi Arabia, which have also reported low levels of knowledge and awareness about HPV and its vaccination. This suggests that the issues identified in our study are not unique to the Medina region and may be prevalent across the country. Therefore, national-level interventions are needed to improve HPV vaccination rates and reduce the incidence of HPV-related diseases in Saudi Arabia.

Saudi Arabia, similar to other developing countries, encounters challenges in controlling HPV and its related health impacts, such as cervical cancer. The consistent rise in the prevalence of cervical cancer underscores the need for effective vaccination programs [21]. Although preventive measures for HPV infection, such as the HPV vaccination, are widely available, vaccine uptake is often hindered by public hesitation and a lack of awareness. Notably, in Saudi Arabia, cultural concerns and insufficient information represent significant barriers to the acceptance of HPV vaccination [22]. Hence, understanding public knowledge and attitudes toward HPV and its vaccination is essential for developing customized health education and vaccination campaigns that can effectively overcome these barriers and improve vaccine adoption [23].

The lower knowledge and awareness concerning HPV and its vaccination among women in Medina could be attributed to several factors, including limited access to accurate information and the influence of social norms. Our study found that social media and the internet were the primary sources of information for many participants, which may contribute to the spread of misinformation. Additionally, the significant association between higher levels of education and better knowledge and awareness suggests that educational interventions could effectively improve awareness and vaccination rates. Also, the lower perception of HPV risk in Medina due to its strict religious nature compared to the other regions could lead to reduced motivation to acquire information regarding HPV [24]. Consequently, individuals who do not see themselves at risk for HPV infection might be less motivated to gain knowledge about HPV vaccination.

Islamic countries have a unique sociocultural framework in which religious beliefs and actions can notably influence social norms. It is well-known that in Islam, individuals must abstain from sex until they get married. As HPV is transmitted sexually, some parents may consider vaccination for their daughters unnecessary or immoral, believing that it could encourage premature sexual behavior [25]. Nevertheless, in our study, a few participants (5.4%) reported that cultural or religious beliefs represented the main barrier preventing them from taking the HPV vaccine, and another 51 (7.1%) reported family refusal as the primary barrier to vaccination.

Significantly, 419 participants reported the lack of awareness about HPV and its vaccination as a primary reason preventing them from receiving the HPV vaccine. In the same context, Radwan et al. highlighted a significant association between the knowledge and awareness of HPV and the willingness to receive the HPV vaccine [26]. Therefore, there is a need to improve public awareness about HPV and its vaccination to enhance the vaccination rates among the population. The cost of vaccination could also represent a vaccination barrier, as our study reported that around 4.2% of the participants reported the cost of the vaccine as a potential barrier to HPV vaccination. This was supported by Turki et al., who found that most respondents were willing to be vaccinated against HPV if it was provided at no cost by the healthcare facilities [18]. As a result, we encourage healthcare facilities to offer the HPV vaccine at no cost or at least at a lower cost, particularly for poor regions.

The HPV vaccine is highly effective at preventing infections with HPV types that are responsible for approximately 90% of cervical cancers, as well as other cancers such as those of the anus, penis, vulva, vagina, and oropharynx. Studies have shown that HPV vaccination can prevent more than 90% of HPV-related cancers when administered at the recommended ages [27]. However, as with many other vaccines, the HPV vaccine has mild side effects, such as soreness, redness, arm swelling, fever, nausea, and muscle or joint pain [28]. Our study found that around 29.4% of the participants were concerned about vaccine safety as a potential barrier to vaccine administration. Radwan et al. also reported a similar percentage (27.7%) of participants who are not willing to administrate the HPV vaccine as they have concerns about its potential side effects [26]. Healthcare care providers should address the concerns about the HPV side effects of the HPV vaccine and better clarify for the patients that these side effects are mild and should not be a barrier preventing them from receiving the vaccine.

Our study also reported that fear of injections represents a significant barrier to HPV vaccination, as 17.5% of the participants reported it as a potential barrier. This was slightly higher than that of Radwan et al., who reported that 8.56% of the participants reported the fear of injection as a reason for not receiving the vaccine [26]. Moreover, Turki et al. reported a slightly higher percentage of participants (27.7%) as a potential barrier to not vaccinating [18]. The variety of health education, level of education, and average age of participants could explain these minor differences between studies. Fear of injection should not be considered a significant barrier, and clinicians should address this issue and make people aware that fear of injections should not be a barrier to vaccination. Furthermore, future researchers should focus on developing new routes for vaccine administration to increase vaccination rates.

Social media (35.1%) and the internet (30.8%) were considered the main primary resources regarding the HPV vaccine in our study, with a smaller percentage of the population (26.6%) reporting doctors and health professionals as the primary sources of information. This was consistent with a previous cross-sectional study conducted in Mekka, which found that the internet and social media represent 48% of the primary sources of HPV infection and its vaccination [18]. This notable high percentage highlights the increasing role of online platforms in acquiring health information. Still, it also underscores the risk of misinformation and the need to provide reliable and accurate information to the public.

Our study also reported a significant association between the level of education (*p* < 0.01) or monthly income (*p* < 0.0001) and the knowledge and awareness of HPV and its vaccination. This aligned with Algaadi et al., Radwan et al., and Turki et al. [15,18,26]. Algaadi et al. also found that healthcare professionals showed significantly higher knowledge about HPV infection and its vaccines than individuals in other occupations [15]. These findings support our recommendation that more health education is required to increase awareness and knowledge about HPV and its vaccines.

Also, our findings indicate significant associations between educational status and monthly income with knowledge and awareness about HPV and its vaccination. This is consistent with Radwan et al., who also found an association with the level of education. However, Turki et al. identified additional factors such as age, nationality, marital status, number of children, and occupation as significantly associated with HPV vaccination knowledge [18,26]. These discrepancies may be attributed to differences in study design, sample size, and regional variations. For instance, our study focused specifically on women in the Medina region, which may have unique cultural, social, and economic characteristics influencing knowledge and awareness about HPV and its vaccination. The differences in demographic composition between the study populations could also contribute to the observed variations. These findings suggest that unique, community-specific interventions may be required to improve knowledge about HPV and its vaccination. Tailoring interventions to address the specific needs and barriers of different communities can enhance their effectiveness. For example, in regions where cultural or religious beliefs significantly influence health behaviors, culturally sensitive educational campaigns and community engagement initiatives may be necessary to promote HPV vaccination.

Compared to the general population, our study participants had a higher level of education and were predominantly young and single. According to the General Authority for Statistics in Saudi Arabia, the literacy rate among Saudi women is around 91%, and the majority of the population is under 30 years old. However, the national average for higher education attainment is lower than that observed in our study, indicating that our sample may have better access to educational resources.

Moreover, our findings revealed that 59.4% of participants had heard about HPV, and only 37% knew that it was sexually transmitted. This is consistent with previous studies in Saudi Arabia, such as Algaadi et al., who reported that 56.3% of participants had poor knowledge regarding HPV vaccines [15]. Similarly, a study by Radwan et al. in Jeddah found that only 35.9% of participants knew that HPV is sexually transmitted [26]. These findings highlight a significant gap in knowledge and awareness about HPV among Saudi women.

Finally, around 40% of our participants showed a positive attitude toward receiving the HPV vaccine, which aligns with the findings of Turki et al., who reported that 41.5% of participants were willing to receive the vaccine if it was provided at no cost [18]. The main barriers identified in our study were a lack of awareness (57.3%), concerns about vaccine safety (29.4%), and fear of needles (17.5%). These barriers are similar to those reported in other studies, such as the one by Alnaeem et al., which found that cultural and religious beliefs significantly influenced vaccination attitudes [16]. Moreover, our findings align with those from other Islamic countries, where similar barriers to HPV vaccination have been identified. For instance, a scoping review by Kisa and Kisa (2024) highlighted that vaccine hesitancy in Islamic countries is often influenced by religious beliefs, misconceptions about the vaccine’s safety and necessity, and concerns about its compatibility with religious norms [24]. Additionally, a previous systematic review found moderate to high HPV vaccine acceptability despite low to moderate knowledge about HPV infection among several sub-populations in Arab countries [29]. These findings are consistent with our study, where a significant proportion of participants were willing to receive the HPV vaccine despite having limited knowledge about HPV. By comparing our results with these studies, we can better understand the common barriers and facilitators for HPV vaccine uptake in Islamic countries and develop more effective, culturally sensitive interventions.

Our study encompasses multiple strengths. First, our sample size was relatively high, thus ensuring the statistical significance of our data and its applicability to the whole population. Our study also included a diverse population with differences in educational status, occupation, marital status, and monthly income, supporting our results’ generalizability to the whole population. As with any research present, our study was not free of limitations. First, the employment of self-reported data could introduce bias. Second, the cross-sectional design of our study limits the establishment of causality.

This cross-sectional study updates the available literature regarding the knowledge, awareness, and attitudes towards HPV and its vaccination within the Medina population. The findings highlight a notable gap in public knowledge, with only 59.4% of participants knowing about HPV and a lower percentage of participants understanding its mode of transmission and potential health outcomes. The study also highlights social media and internet resources as the primary resources for information regarding HPV and its vaccine, indicating the increasing influence of electronic platforms in health education. Additionally, our analysis showed that education and monthly income were notably linked to a higher level of awareness, reflecting that socioeconomic variables may play an essential role in knowledge and awareness.

To improve HPV vaccination rates, we recommend the following strategies: 

Educational Campaigns: Implement targeted educational campaigns to raise awareness about HPV and its vaccines. These campaigns should be culturally sensitive and address common misconceptions about HPV and its transmission; 

Healthcare Provider Training: Train healthcare providers to effectively communicate the benefits and safety of HPV vaccines to patients. Providers should be equipped to address concerns about vaccine side effects and the importance of vaccination before sexual activity; 

School-Based Programs: Introduce HPV education and vaccination programs in schools to reach adolescents before they become sexually active. Studies have shown that school-based programs are effective in increasing vaccination rates; 

Subsidized Vaccination Programs: Offer the HPV vaccine at reduced or no cost, particularly in low-income areas. Financial barriers are a significant deterrent to vaccination, as highlighted by Turki et al. [18]; 

Use of Social Media: Leverage social media platforms to disseminate accurate information about HPV and its vaccines. Given that 35.1% of our participants cited social media as their primary source of information, this approach could significantly enhance awareness.

Future research should give more attention to developing alternative approaches for HPV vaccine administration to address the fear of injection indicated by some people in our study. Future researchers should also investigate the role of cultural and religious beliefs in forming attitudes toward HPV vaccination to create culturally sensitive educational campaigns. Additionally, longitudinal studies that assess changes in knowledge and awareness regarding HPV vaccination could give us valuable insights concerning the long-term effectiveness of public health approaches.

## 5. Conclusions

Our cross-sectional analytical study revealed a significant gap in knowledge and awareness about HPV and its vaccines among Saudi women in the Medina region. Despite the implementation of HPV vaccination in the national immunization program, uptake remains suboptimal due to various barriers, including lack of awareness, concerns about vaccine safety, and fear of needles. Higher levels of education and income were associated with better knowledge and awareness, highlighting the role of socioeconomic factors. To improve vaccination rates, we recommend targeted educational campaigns, healthcare provider training, school-based programs, subsidized vaccination, and leveraging social media for information dissemination. Future research should focus on longitudinal studies to assess changes in knowledge and awareness, explore cultural and religious influences, and investigate alternative vaccine administration routes. Addressing these barriers is crucial for reducing the incidence of HPV-related diseases and promoting better health outcomes for Saudi women.

## Figures and Tables

**Table 1:** Characteristics of the included participants.

Variables		N (%)
**Age groups**	18–25 years	326 (45.2%)
	26–30 years	77 (10.7%)
	31–35 years	62 (8.6%)
	36–40 years	69 (9.6%)
	41–45 years	60 (8.3%)
	46–50 years	63 (8.7%)
	More than 50 years	64 (8.9%)
**Marital status**		
	Single	370 (51.3%)
	Married	299 (41.5%)
	Divorced	41 (5.7%)
	Widow	11 (1.5%)
**Nationality**		
	Saudi	686 (95.1%)
	Non-Saudi	35 (4.9%)
**Level of Education**		
	Less than high school	15 (2.1%)
	Secondary Education	141 (19.5%)
	Diploma	25 (3.5%)
	Bachelor’s degree	490 (68.0%)
	Master’s degree	41 (5.7%)
	Doctoral degree	9 (1.2%)
**Occupational status**		
	Governmental sector	154 (21.4%)
	Private sector	71 (9.8%)
	Retired	32 (4.4%)
	Student	274 (38.1%)
	Self-employed	34 (4.7%)
	Unemployed	156 (21.6%)
**Region**		
	Al-Madinah	690 (95.8%)
	Yanbu	19 (2.6%)
	Badr	3 (0.4%)
	Al-Ula	1 (0.1%)
	Al-Hanakiyah	3 (0.4%)
	Mahd Adh Dhahab	2 (0.3%)
	Khaybar	3 (0.4%)
**Monthly family income**		
	Less than 3000 RS/month	159 (22.1%)
	3000–10,000 RS/month	243 (33.6%)
	10,000–20,000 RS/month	227 (31.5%)
	More than 20,000 RS/month	92 (12.8%)

**Table 2:** Knowledge and awareness of participants regarding HPV and its vaccination.

Variables		N (%)
**Have you ever heard about HPV?**		
	Yes	428 (59.4%)
	No	293 (40.6%)
**HPV is a virus that is sexually transmitted.**		
	Yes	267 (37.0%)
	No	82 (11.4%)
	I do not know	372 (51.6%)
**HPV will usually go away on its own without treatment.**		
	Yes	45 (6.2%)
	No	227 (31.5%)
	I do not know	449 (62.3%)
**HPV causes cervical cancer.**		
	Yes	270 (37.4%)
	No	29 (4.0%)
	I do not know	422 (58.6%)
**What are your primary sources for information and recommendations about the HPV vaccine?**		
	Social media	253 (35.1%)
	Friends	37 (5.1%)
	Family	17 (2.4%)
	Internet	222 (30.8%)
	Doctor and health professionals	192 (26.6%)
**Have you ever heard about the HPV vaccine?**		
	Yes	392 (54.4%)
	No	329 (45.6%)
**Is the HPV vaccine effective against HPV?**		
	Yes	300 (41.6%)
	No	18 (2.5%)
	I do not know	403 (56.9%)
**Does human papillomavirus vaccine prevent cervical cancer?**		
	Yes	236 (32.7%)
	No	34 (4.7%)
	I do not know	451 (62.6%)
**Is the HPV vaccine available in your country?**		
	Yes	343 (47.5%)
	No	17 (2.4%)
	I do not know	361 (50.1%)
**Can the HPV vaccine cause side effects?**		
	Yes	130 (18.0%)
	No	40 (5.5%)
	I do not know	551 (76.5%)
**Can the HPV vaccine cause HPV infection?**		
	Yes	53 (7.4%)
	No	191 (26.5%)
	I do not know	477 (66.1%)
**Does the HPV vaccine decrease the chance of having changes in the Pap smear?**		
	Yes	155 (21.5%)
	No	37 (5.1%)
	I do not know	529 (73.4%)
**Do females need to be screened for HPV before being vaccinated?**		
	Yes	354 (49.1%)
	No	67 (9.3%)
	I do not know	300 (41.6%)
**Do males need to be screened for HPV before being vaccinated?**		
	Yes	218 (30.2%)
	No	93 (12.9%)
	I do not know	410 (56.9%)
**Do you know the schedule for a human papillomavirus vaccine?**		
	Yes	130 (18.0%)
	No	353 (49.0%)
	I do not know	238 (33.0%)
**Which group should be vaccinated?**		
	Women	234 (32.5%)
	Men	7 (1.0%)
	I do not know	221 (30.7%)
	Both	259 (35.8%)
**Have you been vaccinated for HPV?**		
	Yes	22 (3.1%)
	No	699 (96.9%)
**Are you willing to receive the HPV vaccine, which can protect against HPV infection?**		
	Yes	461 (63.9%)
	No	260 (36.1%)
**Would you recommend the HPV vaccine for a child or adolescent (aged between 9 and 12 years old)?**		
	Yes	262 (36.3%)
	No	459 (63.7%)
**Would you recommend the HPV vaccine for a friend or relative?**		
	Yes	488 (67.7%)
	No	233 (32.3%)

**Table 3:** Attitude toward HPV vaccination among participants.

Attitude		N (%)
**HPV vaccine is effective at preventing cervical cancer.**		
	Strongly disagree	18 (2.5%)
	Disagree	30 (4.2%)
	Neutral	308 (42.7%)
	Agree	188 (26.1%)
	Strongly agree	177 (24.5%)
**I will take the vaccine because I feel at risk of contracting HPV.**		
	Strongly disagree	41 (5.7%)
	Disagree	99 (13.7%)
	Neutral	297 (41.2%)
	Agree	160 (22.2%)
	Strongly agree	124 (17.2%)
**Having only one sex partner can protect from HPV infection.**		
	Strongly disagree	19 (2.6%)
	Disagree	82 (11.4%)
	Neutral	327 (45.3%)
	Agree	162 (22.5%)
	Strongly agree	131 (18.2%)
**It is not necessary to receive the human papillomavirus vaccination.**		
	Strongly disagree	63 (8.7%)
	Disagree	184 (25.5%)
	Neutral	311 (43.2%)
	Agree	88 (12.2%)
	Strongly agree	75 (10.4%)
**I believe that the side effects of the vaccine are reasonable and will not prevent me from taking the vaccine.**		
	Strongly disagree	34 (4.7%)
	Disagree	50 (6.9%)
	Neutral	351 (48.7%)
	Agree	185 (25.7%)
	Strongly agree	101 (14.0%)
**I feel it is better to be vaccinated before becoming sexually active.**		
	Strongly disagree	24 (3.3%)
	Disagree	41 (5.7%)
	Neutral	317 (44.0%)
	Agree	186 (25.8%)
	Strongly agree	153 (21.2%)
**More information on HPV and its vaccine will be needed before I take the vaccine.**		
	Strongly disagree	12 (1.7%)
	Disagree	21 (2.9%)
	Neutral	182 (25.2%)
	Agree	144 (20.0%)
	Strongly agree	362 (50.2%)
**The human papillomavirus vaccine may have long-term negative effects.**		
	Strongly disagree	29 (4.0%)
	Disagree	112 (15.5%)
	Neutral	390 (54.2%)
	Agree	112 (15.5%)
	Strongly agree	78 (10.8%)
**I feel that only sexually active women should receive the vaccine.**		
	Strongly disagree	50 (6.9%)
	Disagree	143 (19.8%)
	Neutral	364 (50.5%)
	Agree	85 (11.8%)
	Strongly agree	79 (11.0%)
**My parents would not allow me to receive the vaccine.**		
	Strongly disagree	75 (10.4%)
	Disagree	153 (21.2%)
	Neutral	350 (48.6%)
	Agree	76 (10.5%)
	Strongly agree	67 (9.3%)
**Education on HPV should be implemented at school.**		
	Strongly disagree	9 (1.2%)
	Disagree	27 (3.7%)
	Neutral	177 (24.5%)
	Agree	163 (22.7%)
	Strongly agree	345 (47.9%)
**HPV vaccination should be included on the national program on immunization.**		
	Strongly disagree	21 (2.9%)
	Disagree	44 (6.1%)
	Neutral	258 (35.8%)
	Agree	165 (22.9%)
	Strongly agree	233 (32.3%)
**What are the main reasons or barriers that have prevented you from receiving the HPV vaccine?**		
Lack of access to healthcare facilities		74 (10.3%)
Cost of the vaccine		30 (4.2%)
Fear of needles or injections		126 (17.5%)
Concern about vaccine safety		212 (29.4%)
Lack of awareness about HPV and the vaccine		419 (57.3%)
Culture or religious beliefs		39 (5.4%)
Family refusal		51 (7.1%)
No time		110 (15.3%)
Other		13 (1.8%)
None		23 (3.2%)

**Table 4:** Association between participants’ demographics and overall knowledge and awareness level of HPV and HPV vaccination.

Variables		Good Knowledge and Awareness	Poor Knowledge and Awareness	*p*-Value
		N (%)	N (%)	
**Age groups**				
	18–25 years	86 (49.2%)	240 (44.0%)	0.148
	26–30 years	22 (12.6%)	55 (10.1%)	
	31–35 years	14 (8.0%)	48 (8.8%)	
	36–40 years	18 (10.3%)	51 (9.3%)	
	41–45 years	13 (7.4%)	47 (8.6%)	
	46–50 years	6 (3.4%)	57 (10.4%)	
	More than 50 years	16 (9.1%)	48 (8.8%)	
**Marital status**				
	Single	95 (54.3%)	275 (50.3%)	0.463
	Married	71 (40.6%)	228 (41.8%)	
	Divorced	6 (3.4%)	35 (6.4%)	
	Widow	3 (1.7%)	8 (1.5%)	
**Nationality**				
	Saudi	169 (96.6%)	517 (94.7%)	0.313
	Non-Saudi	6 (3.4%)	29 (5.3%)	
**Level of education**				
	Less than high school	3 (1.7%)	12 (2.2%)	**0.002** *
	Secondary Education	28 (16.0%)	113 (20.7%)	
	Diploma	3 (1.7%)	22 (4.0%)	
	Bachelor’s degree	119 (68.0%)	371 (67.9%)	
	Master’s degree	15 (8.6%)	26 (4.8%)	
	Doctoral	7 (4.0%)	2 (0.4%)	
**Occupational status**				
	Governmental sector	43 (24.5%)	111 (20.3%)	0.504
	Private sector	18 (10.3%)	53 (9.7%)	
	Retired	5 (2.9%)	27 (4.9%)	
	Student	70 (40.0%)	204 (37.5%)	
	Self-employed	6 (3.4%)	28 (5.1%)	
	Unemployed	33 (18.9%)	123 (22.5%)	
**Region**				
	Al-Madinah	172 (98.3%)	518 (94.9%)	0.660
	Yanbu	2 (1.1%)	17 (3.1%)	
	Badr	1 (0.6%)	2 (0.4%)	
	Al-Ula	0 (0.0%)	1 (0.2%)	
	Al-Hanakiyah	0 (0.0%)	3 (0.5%)	
	Mahd Adh Dhahab	0 (0.0%)	2 (0.4%)	
	Khaybar	0 (0.0%)	3 (0.5%)	
**Monthly family income**				
	Less than 3000 RS/month	29 (16.6%)	130 (23.8%)	**<0.0001** *
	3000–10,000 RS/month	47 (26.9%)	196 (35.9%)	
	10,000–20,000 RS/month	54 (30.8%)	173 (31.7%)	
	More than 20,000 RS/month	45 (25.7%)	47 (8.6%)	

* *p*-value < 0.05 indicates a significant association.

## Data Availability

Data are available upon request.

## References

[1] Ba W. (2006). Epidemiology and natural history of genital human papillomavirus infection. J. Am. Osteopath. Assoc..

[2] Okunade K.S. (2020). Human papillomavirus and cervical cancer. J. Obstet. Gynaecol..

[3] Trottier H., Franco E.L. (2006). The epidemiology of genital human papillomavirus infection. Vaccine.

[4] de Martel C., Plummer M., Vignat J., Franceschi S. (2017). Worldwide burden of cancer attributable to HPV by site, country and HPV type. Int. J. Cancer.

[5] Arbyn M., Weiderpass E., Bruni L., De Sanjosé S., Saraiya M., Ferlay J., Bray F. (2020). Estimates of incidence and mortality of cervical cancer in 2018: A worldwide analysis. Lancet Glob. Health.

[6] Zhang S., Xu H., Zhang L., Qiao Y. (2020). Cervical cancer: Epidemiology, risk factors and screening. Chin. J. Cancer Res..

[7] Farahat F.M., Faqih N.T., Alharbi R.S., Mudarris R.I., Alshaikh S.A., Al-Jifree H.M. (2021). Epidemiological characteristics of cervical cancer in a tertiary care hospital, western Saudi Arabia: A retrospective record-based analysis from 2002–2018. Saudi Med. J..

[8] Obeid D., Alsuwairi F., Alnemari R., Al-Qahtani A., Kurdi W., Alfareh M., Alsanea M., Alabdulkareem M., Alharbi L., Alhamlan F.S. (2024). Sexually transmitted infections in the middle east and North Africa: Comprehensive systematic review and meta-analysis. BMC Infect Dis..

[9] Chan S.S.C., Yan Ng B.H., Lo W.K., Cheung T.H., Hung Chung T.K. (2009). Adolescent girls’ attitudes on human papillomavirus vaccination. J. Pediatr. Adolesc. Gynecol..

[10] Markowitz L.E., Dunne E.F., Saraiya M., Lawson H.W., Chesson H., Unger E.R. (2007). Centers for Disease Control and Prevention (CDC); Advisory Committee on Immunization Practices (ACIP). Quadrivalent Human Papillomavirus Vaccine: Recommendations of the Advisory Committee on Immunization Practices (ACIP). MMWR Recomm. Rep..

[11] Bruni L., Diaz M., Barrionuevo-Rosas L., Herrero R., Bray F., Bosch F.X., de Sanjosé S., Castellsagué X. (2016). Global estimates of human papillomavirus vaccination coverage by region and income level: A pooled analysis. Lancet Glob. Health.

[12] Human Papillomavirus: Vaccine Preventable Diseases Surveillance Standards. https://www.who.int/publications/m/item/vaccine-preventable-diseases-surveillance-standards-hpv.

[13] Darraj A.I., Arishy A.M., Alshamakhi A.H., Osaysi N.A., Jaafari S.M., Sumayli S.A., Mushari R.Y., Alhazmi A.H. (2022). Human Papillomavirus Knowledge and Vaccine Acceptability in Jazan Province, Saudi Arabia. Vaccines.

[14] Saudi Food & Drug Authority (2016). National Manual for Surveillance of Adverse Events Following Immunization in Saudi Arabia. https://old.sfda.gov.sa/ar/drug/resources/DocLib2/Drug-resoyrce-2143.

[15] Algaadi S.A., Aldhafiri H.J., Alsubhi R.S., Almakrami M., Aljamaan N.H., Almulhim Y.A. (2024). The Saudi Population’s Knowledge and Attitude Towards Human Papillomavirus (HPV) Infection and Its Vaccination. Cureus.

[16] Alnaeem L., Alanizi S., AlQarni G., Alwadani J., Bomouzah F., Ali Z. (2023). Acceptance, Knowledge, and Attitude of Parents Toward the Human Papillomavirus Vaccine in the Eastern Region of Saudi Arabia: A Cross-Sectional Study. Cureus.

[17] Almaghlouth A.K., Bohamad A.H., Alabbad R.Y., Alghanim J.H., Alqattan D.J., Alkhalaf R.A., Almaghlouth A.K., Bohamad A.H., Alabbad R.Y., Alghanim J.H. (2022). Acceptance, Awareness, and Knowledge of Human Papillomavirus Vaccine in Eastern Province, Saudi Arabia. Cureus.

[18] Turki Y.M., Alqurashi J. (2023). Knowledge, Attitudes, and Perceptions Towards Human Papillomavirus (HPV) Vaccination Among Adult Women in Primary Health Care Centers in Makkah, Saudi Arabia. Cureus.

[19] Alkalash S.H., Alshamrani F.A., Alamer E.H.A., Alrabi G.M., Almazariqi F.A., Shaynawy H.M., Alkalash S.H., Alshmrani F., Alamer E.H.A., Alrabi G.M. (2022). Parents’ Knowledge of and Attitude Toward the Human Papillomavirus Vaccine in the Western Region of Saudi Arabia. Cureus.

[20] Cuschieri S. (2019). The STROBE guidelines. Saudi J. Anaesth..

[21] Alhamlan F.S., Khayat H.H., Ramisetty-Mikler S., Al-Muammar T.A., Tulbah A.M., Al-Badawi I.A., Kurdi W.I., Tulbah M.I., Alkhenizan A.A., Hussain A.N. (2016). Sociodemographic characteristics and sexual behavior as risk factors for human papillomavirus infection in Saudi Arabia. Int. J. Infect. Dis..

[22] Tobaiqy M.A., Mehdar S.A., Altayeb T.I., Saad T.M., Alqutub S.T. (2023). Parental knowledge, views, and perceptions of human papilloma virus infection and vaccination-cross-sectional descriptive study. J. Fam. Med. Prim. Care.

[23] Heena H., Durrani S., AlFayyad I., Riaz M., Tabasim R., Parvez G., Abu-Shaheen A. (2019). Knowledge, Attitudes, and Practices towards Cervical Cancer and Screening amongst Female Healthcare Professionals: A Cross-Sectional Study. J. Oncol..

[24] Kisa S., Kisa A. (2024). Religious beliefs and practices toward HPV vaccine acceptance in Islamic countries: A scoping review. PLoS ONE.

[25] Shelton R.C., Snavely A.C., Jesus M.D., Othus M.D., Allen J.D. (2013). HPV Vaccine Decision-Making and Acceptance: Does Religion Play a Role?. J. Relig. Health.

[26] Radwan A., Sabban H., Alsobhi R., Alsayed N., Alharthi T., Alzanbaqi M. (2023). Awareness and Knowledge of Human Papillomavirus (HPV) Infection and Vaccine Among Women: A Cross-Sectional Study in Jeddah, Saudi Arabia. Cureus.

[27] DeKloe J., Urdang Z.D., Martinez Outschoorn U.E., Curry J.M. (2024). Effects of HPV vaccination on the development of HPV-related cancers: A retrospective analysis of a United States-based cohort. J. Clin. Oncol..

[28] Everything You Need to Know About the HPV Vaccine. https://www.gavi.org/vaccineswork/everything-you-need-know-about-hpv-vaccine.

[29] Gamaoun R. (2018). Knowledge, awareness and acceptability of anti-HPV vaccine in the Arab states of the Middle East and North Africa Region: A systematic review. East Mediterr Health J..

